# Therapeutic Potential of Linearol in Combination with Radiotherapy for the Treatment of Glioblastoma In Vitro

**DOI:** 10.3390/ijms24043760

**Published:** 2023-02-13

**Authors:** Vasiliki Zoi, Theodora Papagrigoriou, Olga S. Tsiftsoglou, George A. Alexiou, Maria Giannakopoulou, Eftychia Tzima, Pericles Tsekeris, Anastasia Zikou, Athanasios P. Kyritsis, Diamanto Lazari, Vasiliki Galani

**Affiliations:** 1 Neurosurgical Institute, University of Ioannina, 45500 Ioannina, Greece; 2 Department of Anatomy Histology-Embryology, School of Medicine, University of Ioannina, 45110 Ioannina, Greece; 3 Laboratory of Pharmacognosy, School of Pharmacy, Faculty of Health Sciences, Aristotle University of Thessaloniki, 54124 Thessaloniki, Greece; 4 Department of Neurosurgery, University of Ioannina, 45110 Ioannina, Greece; 5 Department of Radiation Oncology, University of Ioannina, 45110 Ioannina, Greece; 6 Department of Radiology, University of Ioannina, 45110 Ioannina, Greece

**Keywords:** glioblastoma, treatment, linearol, radiation

## Abstract

Glioblastoma is one of the most malignant and lethal forms of primary brain tumors in adults. Linearol, a kaurane diterpene isolated from different medicinal plants, including those of the genus Sideritis, has been found to possess significant anti-oxidant, anti-inflammatory and anti-microbial properties. In this study, we aimed to determine whether linearol could exhibit anti-glioma effects when given alone or in combination with radiotherapy in two human glioma cell lines, U87 and T98. Cell viability was examined with the Trypan Blue Exclusion assay, cell cycle distribution was tested with flow cytometry, and the synergistic effects of the combination treatment were analyzed with CompuSyn software. Linearol significantly suppressed cell proliferation and blocked cell cycle at the S phase. Furthermore, pretreatment of T98 cells with increasing linearol concentrations before exposure to 2 Gy irradiation decreased cell viability to a higher extent than linearol or radiation treatment alone, whereas in the U87 cells, an antagonistic relationship was observed between radiation and linearol. Moreover, linearol inhibited cell migration in both tested cell lines. Our results demonstrate for the first time that linearol is a promising anti-glioma agent and further studies are needed to fully understand the underlying mechanism of this effect.

## 1. Introduction

An increase in the incidence of cancer has been observed in recent years [1]. The central nervous system tumors account for approximately 3% of all neoplasms, and the 5-year survival rate remains less than 25% [2]. Among them, glioblastoma (GBM) is the most common and aggressive glioma form and despite surgical procedures and combination treatment with chemotherapy and radiotherapy, the patient’s median survival remains poor, and recurrence is almost always observed [3]. The standard chemotherapeutic agent for the treatment of glioblastoma is temozolomide (TMZ). However, the diffuse infiltrative nature of glioblastoma cells and the existence of the blood–brain barrier (BBB) considerably reduce the efficacy of this drug and thus current therapy only minimally improves the median survival time of patients [4,5]. Thus, there is an imperative need for the development of new treatments that will work as antiglioma agents.

Some natural compounds have been shown to possess significant antitumor properties, mainly by enhancing apoptosis-related pathways in GBM cells [6]. Several studies have reported the antioxidant role and radio/chemosensitizing properties of natural compounds, including curcumin, resveratrol, epigallocatechin, moschamine and caffeine [7,8,9]. Sideritis is a genus of flowering plants that have been used in traditional medicine in the treatment of gastrointestinal complaints, inflammation and rheumatic disorders [10]. Plants from the genus Sideritis occur mainly in the Mediterranean area, as well as in western and central Asia [11]. Different species of this plant possess a variety of health benefits that have been attributed to the presence of important natural compounds, including flavonoids, phenols and terpenes [12]. *Sideritis scardica*, for example, contains a variety of phenolic compounds that have been shown to possess anti-inflammatory, gastroprotective and cytotoxic effects on different cell lines, whereas the plant’s high levels of flavonoid constituents have been related to significant in vitro cytotoxicity against the C6 rat glioma cell line [13].

**Figure 1:**
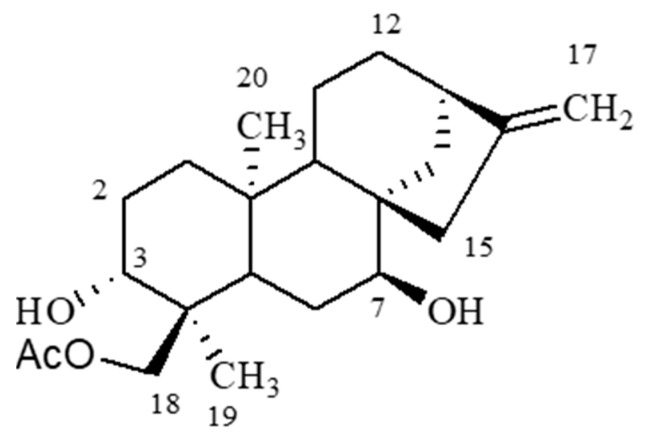
Structure of linearol. AcO: acetoxy group.

Kauranes are a group of tetracyclic diterpenes found in different medicinal plants. The species of the genus Sideritis, in particular, is widely spread in the Mediterranean area and has been identified as the source of important kauranes, including linearol, sidol and foliol [10]. Kaurane diterpenes have been found to possess several beneficial properties, including anti-inflammatory, antimicrobial and antioxidant effects [14,15,16]. Linearol (Figure 1) is a kaurane diterpene with a molecular weight of 362.5 Daltons and thus it is able to cross the blood–brain barrier (BBB). Few studies have investigated the potential health properties of linearol. When this diterpene was obtained as the major compound of the acetone extract of *Sideritis lycia*, it displayed significant antiviral and insecticidal properties. Specifically, linearol isolated from Sideritis species *S. lycia* was investigated for its antiviral, cytotoxic and insecticidal activities. Linearol was obtained as the major compound of the acetone extract of S. lycia (11.3% of the total acetone extract) and was evaluated for its antiviral and cytotoxic potency alone or as part of the acetone extract. Although linearol displayed significant antiviral and insecticidal properties, it failed to induce a satisfactory cytotoxicity when tested on different cancer lines (KB, human epidermoid carcinoma; P-388, mouse leukemia; LNCaP, hormone-dependent human prostate; COL-2, human colon cancer; LU1, human lung cancer; and A2780, human ovarian cancer) at a concentration of 20 μg/mL [17]. Moroever, treatment of activated macrophages with linearol resulted in the regulation of pro-inflammatory pathways, suggesting that linearol may possess anti-inflammatory properties. Specifically, linearol inhibited the release of early pro-inflammatory cytokines, such as tumor necrosis factor a (TNFa) [18]. However, no previous studies have investigated the potential anti-proliferative role of linearol in cancer cells, such as GBM. Here, we investigated the antitumor effects of linearol, alone or in combination with radiotherapy in two GBM cell lines.

## 2. Results

### 2.1. Linearol Inhibits GBM Cell Proliferation and Viability

**Figure 2:**
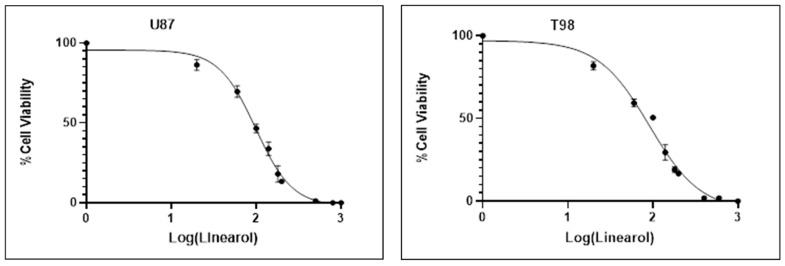
Linearol inhibits T98 and U87 cell proliferation. Cells were quantified by staining with Trypan Blue at 72 h post-treatment and data shown are the means (±S.D.) from 3 different experiments performed in triplicate. The IC50 values were determined using the non-linear regression analysis model of GraphPad Prism.

**Figure 3:**
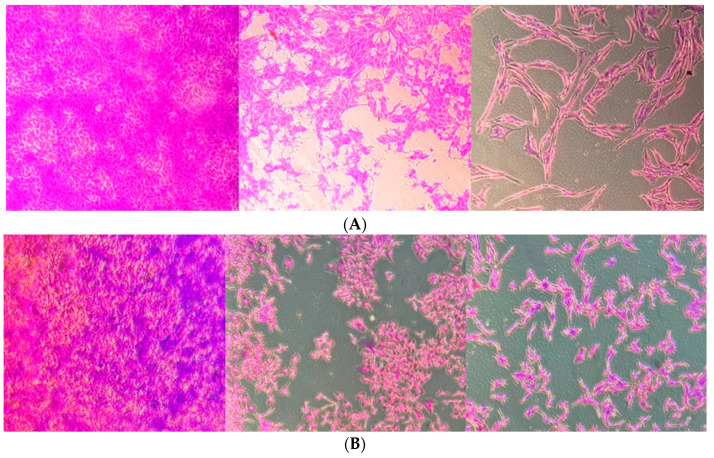
Morphological changes in T98 (**A**) and U87 (**B**) cell lines after staining with 0.2% Crystal Violet Solution. Cells were seeded in 6-well plates; after 24 h two different concentrations of linearol were added and the cells were allowed to proliferate for 72 h. Crystal violet solution was added and the plates were placed on a rocking shaker for 3 min. Excess staining was removed and after washing twice with running water, they were left overnight to dry. Images shown are representative of three different experiments that showed similar results (magnification, 10×). Scale bars = 100 µM.

We investigated the effects of linearol on the viability of two GBM cell lines, U87 and T98, using the Trypan Blue Exclusion Assay. Our results indicate a more pronounced decrease in cell viability at higher concentrations of linearol, suggesting a concentration-dependent inhibitory effect of this agent on both cell lines. The IC50 value of linearol that was determined 72 h post treatment was 98 µM in U87 cells and 91 µM in T98 (Figure 2). Crystal Violet staining was also used to further determine the effects of linearol on cell proliferation and morphology of GBM cells. When U87 and T98 cells were treated with linearol at concentrations of IC50 and 2IC50, changes in the morphology of both cells lines were observed, including cell shrinkage (Figure 3).

### 2.2. Linearol Induces Cell Cycle Arrest

**Figure 4:**
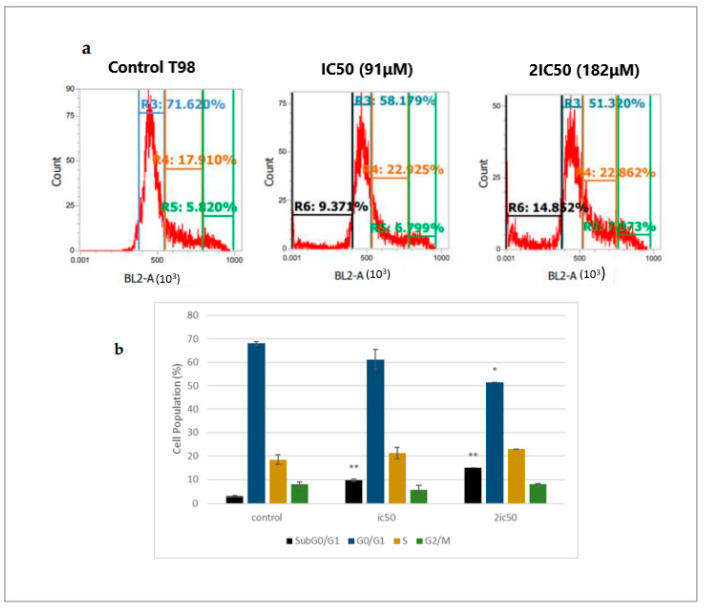
The regulatory effect of linearol on cell cycle distribution in T98 cells. (**a**) Linearol at concentrations of 91 and 182 µM increased the S and SubG0/G1 populations 72 h post-treatment. (**b**) Cell cycle distribution of T98 cells after treatment with linearol and PI staining at 72 h. Cell populations at different cell cycle phases were quantified and data were expressed as mean ± SD from 3 different experiments. * *p* < 0.05; ** *p* < 0.001.

**Figure 5:**
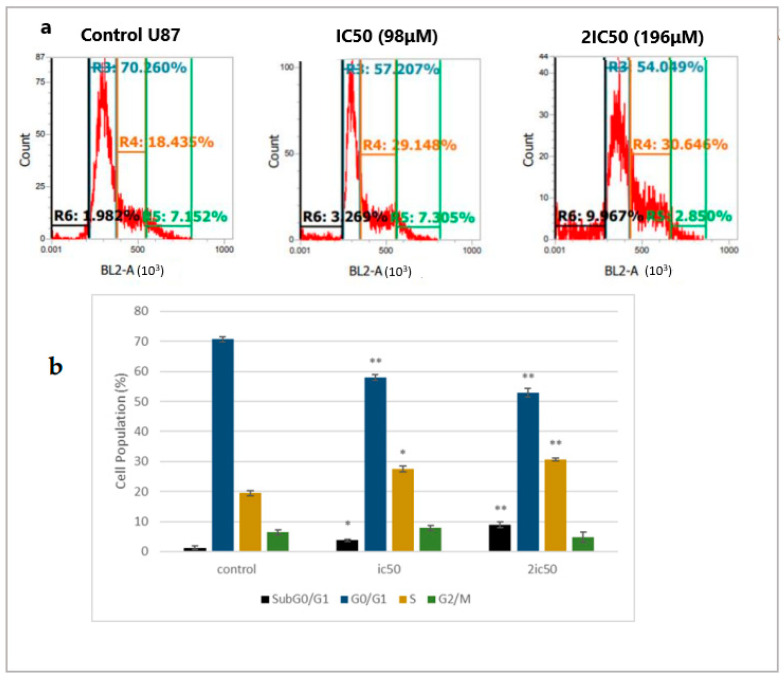
The regulatory effect of linearol on cell cycle distribution in U87 cells. (**a**) Linearol at concentrations of 98 and 196 µM increased the S and SubG0/G1 populations 72 h post-treatment. (**b**) Cell cycle distribution of U87 cells after treatment with linearol and PI staining at 72 h. Cell populations at different cell cycle phases were quantified and data were expressed as mean ± SD from 3 different experiments. * *p* < 0.05; ** *p* < 0.001.

To evaluate whether linearol could cause cell cycle arrest, we used flow cytometry analysis with DNA staining dye, propidium iodide (PI). Cells were treated with IC50 and 2IC50 concentrations for 72 h. Flow cytometry analysis of cell cycle distribution revealed that as the concentration of linearol increased, the number of cells in the S-phase also increased in both T98 (Figure 4a) and U87 (Figure 5a) cell lines. Specifically, when U87 cells were treated with linearol at a concentration of 98 µM, the percentage distribution of cells in the S phase was 27.4% ± 0.98 compared to 19.45% ± 0.88 in the control group. In the T98 cell line, treatment with 182 µM of linearol increased the distribution of cells in the S phase, whereas treatment with 91 or 182 µM of linearol prominently increased the subGO/G1 population compared to the control group (9.7 ± 0.49 and 14.9 ± 0.10, respectively, compared to 2.9 ± 0.81 of control) (Figure 4b). The SubG0/G1 population was also significantly increased in the U87 cell line (3.8 ± 0.39 and 8.9 ± 0.96 for the ic50 and 2ic50 treatment compared to 1.3 ± 0.60 for the control group) (Figure 5b).

### 2.3. Linearol and Radiotherapy Exert Synergistic Anti-Proliferative Results

**Figure 6:**
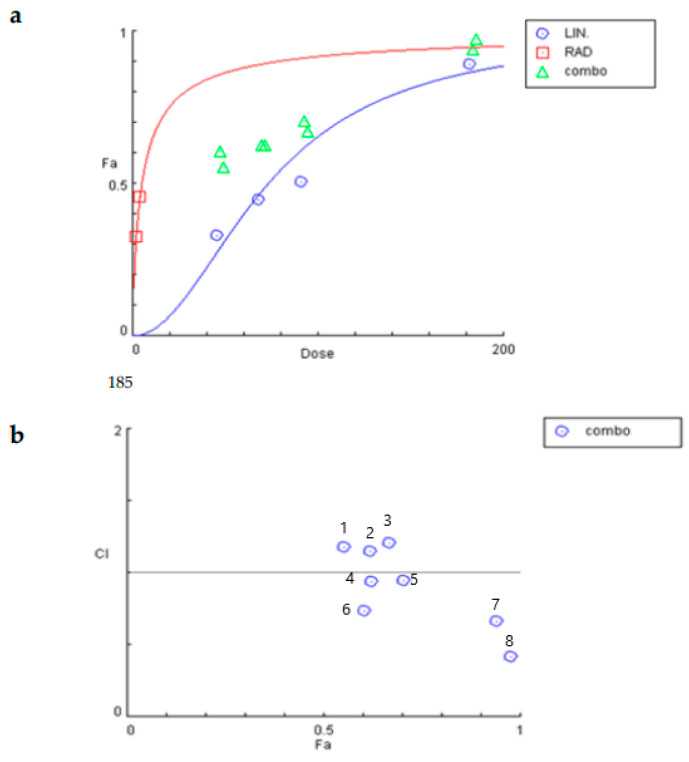
Determination of the synergistic effect of the treatment of linearol with radiation on T98 cells. (**a**) Dose–response curve of linearol and radiotherapy. Combo indicates the different combinations of linearol and radiation. (**b**) Combination index (CI) plot for all combinations. CI = 1 defines additive effect, CI < 1 defines synergism, whereas CI > 1 is antagonism. Numbers refer to different combinations used in the experiment. 1 = Lin 45.5 μΜ + Rad 4 Gy, 2 = Lin 68 μΜ + Rad 4 Gy, 3 = Lin 91 μΜ + Rad 4 Gy, 4 = Lin 68 μΜ + Rad 2 Gy, 5 = Lin 91 μΜ + Rad 2 Gy, 6 = 45.5 μΜ + Rad = 2 Gy, 7 = Lin 182 μΜ + Rad 2 Gy, 8 = Lin 182 μΜ + Rad = 4 Gy.

**Figure 7:**
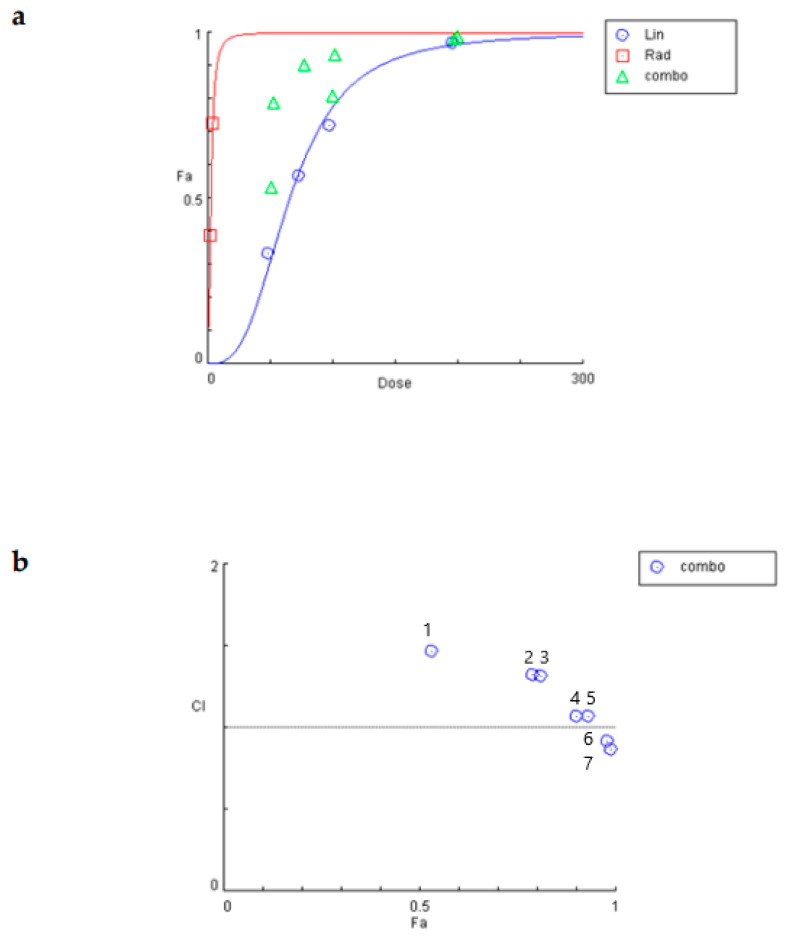
Compusyn analysis for the determination of possible synergistic effect of the treatment of linearol with radiation on U87 cells. (**a**) Dose–response curve of linearol and radiotherapy. Combo indicates the different combinations of linearol and radiation. (**b**) Combination index (CI) plot for all combinations. CI = 1 defines additive effect, CI < 1 defines synergism, whereas CI > 1 is antagonism. Numbers refer to different combinations used in the experiment. 1 = Lin 49 μΜ + Rad 2 Gy, 2 = Lin 98 μΜ + Rad 2 Gy, 3 = Lin 49 μΜ + Rad 4 Gy, 4 = Lin 73.5 μΜ + Rad 4 Gy, 5 = Lin 98 μΜ + Rad 4 Gy, 6 = 196 μΜ + Rad = 2 Gy, 7 = Lin 196 μΜ + Rad 4 Gy.

To determine the synergism or antagonism of the combination of linearol and radiation in T98 or U87 cells, the median-effect equation using CompuSyn software was used. Radiation was given in doses of 2 or 4 Gy. The T98 cell line was selected due to its high resistance to both radiotherapy and chemotherapy. Linearol was used in concentrations ranging from 45.5 to 182 μΜ for the T98 cell line (Figure 6a) and 49 to 196 μΜ for the U87 cells (Figure 7a). The dose–effect curves (Fa-Dose) were created using the % cell mortality results from the Trypan Blue Exclusion Assay for each drug and their combination. In T98 cells, linearol and 2 Gy radiation exhibited a synergistic relationship at all tested combinations, with the higher synergy being observed at a 182 μΜ linearol concentration. The combination of linearol at different concentrations with 4 Gy radiation exhibited mild antagonistic effects, except for the combination of 182 μΜ of linearol and 4 Gy radiation, where the strongest synergistic effect was observed (CI = 0.42) (Figure 6b). In U87 cells, linearol and radiotherapy exhibited an antagonistic relationship in most combinations tested, with the exception of 196 μΜ and 2 or 4 Gy where a synergistic behavior was observed (Figure 7b).

### 2.4. Linearol Inhibits Migration of GBM Cells

**Figure 8:**
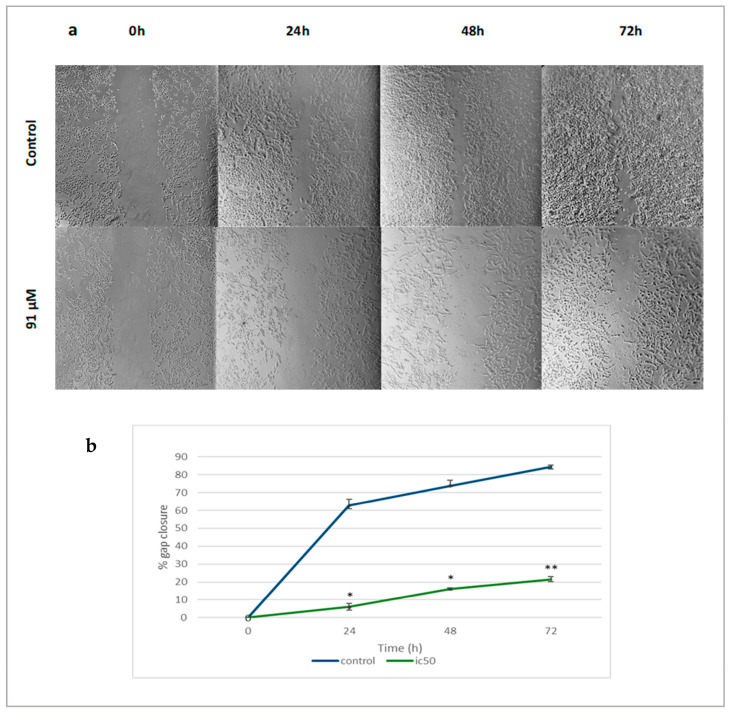
Microscopic images (magnification × 10) of scratch wound healing assay. (**a**) When T98 cells were treated with an IC50 linearol concentration, a decrease in the closure percentage was observed. (**b**) The migration ability of T98 cells showed a statistically significant decline after treatment with linearol when compared to control (91 µM at 24 h: 6.05%, *p* < 0.05; 91 µM at 48 h: 16.06%, *p* < 0.05 and at 72 h: 21.56%, *p* < 0.001). Data are presented as the mean ± SD. * *p* < 0.05, ** *p* < 0.001 compared to the control group.

**Figure 9:**
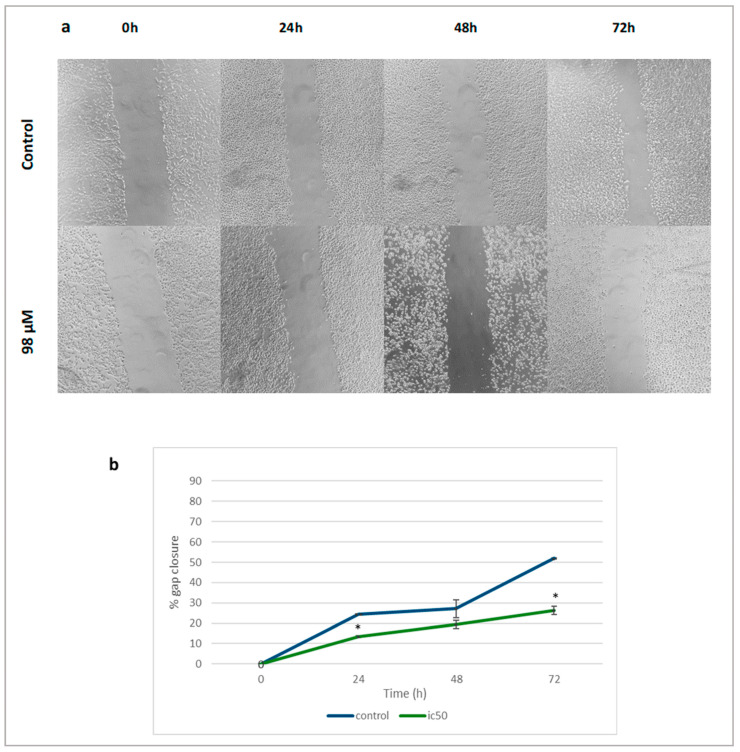
Microscopic images (magnification ×10, scale bars = 100 μΜ) of scratch wound healing assay. (**a**) When U87 cells were treated with an IC50 linearol concentration, a decrease in the closure percentage was observed. (**b**) The migration ability of U87 cells showed a statistically significant decline after treatment with linearol when compared to control (98 µM at 24 h: 13.38%, *p* < 0.05; 98 µM at 72 h: 26.29%, *p* < 0.05). Data are presented as the mean ± SD. * *p* < 0.05 compared to the control group.

The anti-migratory effects of linearol were investigated through scratch wound assay. The migration rate of treated T98 (Figure 8a) and U87 (Figure 9a) cells with increased linearol concentrations was calculated based on the closure of the gap on the monolayer GBM cells. Treatment with IC50 of linearol showed a significant decrease in the percentage of wound closure compared to the control group after 24, 48 and 72 h incubation time. The closure percentage of GBM cells showed significant statistical differences when compared to control for both cell lines. Specifically, in T98 cells, after treatment with 91 µM of linearol, the percentage of wound closure was 6.05% ± 1.76, 16.06% ± 0.56 and 21.56% ± 1.29 after 24, 48 and 72 h of incubation time, respectively, whereas the corresponding percentages for the control group were 62.77% ± 3.62, 73.96% ± 2.86 and 84.42% ± 0.84, respectively (Figure 8b). Similar results were observed for the U87 cell line (Figure 9b), where treatment with 98 µM of linearol resulted in a statistically significant decrease in the migration ability of GBM cells (26.29% ± 2.01 gap closure at 72 h compared to 51.88% ± 0.16 for the control group).

## 3. Discussion

GBM remains the most common primary brain tumor in adults. Since 2005, the Stupp Protocol, which consists of surgical resection followed by TMZ chemotherapy and adjuvant radiation therapy, has been the standard of care [19]. However, treatment of GBM has achieved very limited success in increasing overall survival of patients. Although research has nowadays focused on novel anti-glioma agents, both natural and synthetic, several obstacles related to this specific type of brain tumor have constrained their success. The heterogeneous nature of GBM, the complex interplay among the different components within the tumor microenvironment and the presence of the blood–brain barrier account for the adaptive capacities of GBM and the increased resistance to treatment [20,21].

Multimodal therapy techniques, in which various therapies or therapeutic agents are combined with the aim of achieving a higher cytotoxic effect on tumor cells, can serve as a successful strategy for the treatment of glioblastoma [22]. Recently, many plant-derived compounds have been shown to possess significant anti-tumor properties, mainly by enhancing cell cycle impairment in GBM cells and apoptosis-related pathways [7]. Numerous studies have shown that certain natural compounds have radio/chemosensitizing properties when combined with radiotherapy and chemotherapy [23,24,25,26,27,28,29,30,31,32,33,34]. Most importantly, some of them can easily cross the BBB, which remains one of the most crucial considerations when developing new therapeutic agents for brain tumors [25].

Among the most well-studied families of plants for the treatment or prevention of different diseases is the Lamiaceae family. The Lamiaceae family consists of around 7200 species of medicinal plants distributed in 240 genera [26]. Plants of this family are indigenous to Mediterranean regions, including Turkey, Spain and Greece, and have traditionally been used for several medicinal purposes [27,28]. Nowadays, plants from the Lamiaceae family are known for their biological activities, including antioxidant, antiviral and antibacterial [29]. The World Health Organization has stated that plants of this family have a long history of use and proven therapeutic effects, and thus they should be further investigated for new medicinal properties that could make them promising candidates for the prevention or treatment of new diseases [30]. In this study, we examined the possible antiglioma effects of linearol, a kaurane diterpene of the Lamiaceae family, and specifically of the genus Sideritis alone or in combination with radiotherapy, which remains an important constituent part of standard therapy.

This study showed for the first time that linearol is a promising natural compound for the treatment of GBM in vitro. Linearol inhibited cell proliferation and migration of two malignant glioma cell lines, U87 and T98. For most of our experiments, we focused on two concentrations of linearol, IC50 and 2IC50. The choice of using a higher concentration (2IC50) was made so as to highlight the strong dose-dependent anti-proliferative effects of linearol. Linearol also induced cell cycle arrest and decreased cell viability in both cell lines. The most prominent effect on cell cycle was observed in U87 cells, where linearol induced S-arrest. On T98 cells, no significant effect on cell cycle arrest was observed, and that may be attributed to the fact that these cells are known to be more resistant to different chemotherapeutic approaches, including the approved drug Temozolomide. In fact, different studies have shown that temozolomide has an IC50 exceeding 1000 μM in T98 cells [31,32,33]. When linearol was combined with 2 Gy irradiation, its anti-proliferative effects were further enhanced in T98 cells in all tested combinations and only in two combinations in the U87 cell line. Since GBM is difficult to cure by neurosurgery or radiotherapy alone, and T98 in particular is a line generally resistant to both radiation and chemotherapy, the combination of linearol and radiation could be a potentially promising treatment, particularly for resistant tumors.

There are no previous studies that have explored the potential anti-glioma effects of linearol. In a recent study, Castrillo et al., investigated the effects of linearol on apoptosis and phagocytosis. When macrophages challenged with bacterial lipopolysaccharide (LPS) were exposed to increasing linearol concentrations, the activation of nuclear factor kB (NF-kB) was inhibited. Since this transcription factor is a central mediator of apoptosis and immune response, it was surmised that linearol can protect activated macrophages from apoptosis. In the same study, linearol inhibited the expression of NOS-2 and COX-2, as well as the release of tumor necrosis factor-a (TNF-a), thus resulting in the regulation of pro-inflammatory pathways and prolonging of viability of the activated macrophages [18]. The NF-κB signaling pathway is closely related to central aspects of the malignant phenotype of GBM, including rapid proliferation, resistance to chemotherapy and induction of stem-like traits [34,35]. In several studies, constitutive NF-κB activation was observed in primary cultures derived from GBMs [36,37]. Therefore, compounds targeting this pathway, such as linearol, may present useful treatment options and further studies are needed to better understand the molecular mechanism underlying the anti-glioma effect of linearol we have reported in this study.

Radiotherapy remains an important aspect of glioblastoma treatment; however, until today, it has not been thoroughly investigated in combination with potential antiglioma agents [38]. Since linearol showed some promising antiproliferative effects in two human glioma lines, we investigated whether its effects were further enhanced in the presence of irradiation. For the most radio-resistant T98 cell line, a combination of different linearol concentrations with 2 Gy irradiation revealed a synergistic effect, whereas for the U87 cells, an antagonistic effect was observed in almost all given combinations. Since there are no previous studies that have investigated the possible synergistic effects of linearol and radiotherapy, and taking into account that radio-resistance is a principal obstacle to successful GBM treatment, further consideration is needed. A mechanism of radio and chemo-resistance in GBM is linked to distorted redox homeostasis. In particular, GBM radio-resistance has been related to an increased production of ROS scavengers following mitochondrial alterations. This leads to the induction of survival mechanisms, and a significant upregulation of mitochondrial enzymes, like superoxide dismutase (SOD-2) [39,40]. Subsequently, an increase in the level of reduced glutathione is observed, leading to further resistance and increased relapses [41]. Linearol has been found to reduce H2O2-induced oxidative stress in the human astrocytoma U373-MG cell line, and thus protect cells from excessive ROS production. When those cells were pretreated with linearol (5 and 10 μM, 24 h) prior to H2O2 exposition, the levels of intracellular reactive oxygen species were significantly decreased, and the protein expression of antioxidant enzymes was restored. This effect was partly attributed to the activation of the Nrf2 pathway and subsequently antioxidant enzyme induction [42,43].

The present study has several limitations. The pharmacokinetic profile of linearol has yet to be investigated. Thus, there is no information about plasma or intratumoral concentrations of this compound after administration. However, the blood–brain barrier permeability characteristics of linearol have been explored by Burgos et al., and the results suggest that linearol may predominantly move across the blood–brain barrier by passive diffusion [44]. That, in combination with the low molecular weight of this compound there are promising indications of its efficacy and bioavailability. Studies in glioma xenograft models are required to further analyze the full spectrum of the pharmacokinetic profile of this compound. Furthermore, evaluating the combined actions of linearol and radiation 72 h post-treatment, preliminary and additional experiments may be required, including a colony-forming assay [45]. Thus, further experiments are needed to fully understand the mechanism underlying the anti-glioma action of linearol, as well as the causes of the synergistic antiproliferative effects of linearol and irradiation on the radio-resistant T98 human glioma cell line. 

In summary, this study is the first to investigate the possible anti-glioma effects of linearol in vitro alone or in combination with radiation. Our results show that linearol can inhibit cell proliferation of human GBM cells, induce cell cycle arrest and slow down migration. Given that linearol can be isolated from different Sideritis species, most of which are indigenous to Mediterranean countries, including Greece, it can be obtained in sufficient quantities that allow for further investigation. Glioblastoma is a highly heterogenic and infiltrative tumor and recurrence is almost always observed; thus, the need for novel and effective anti-glioma compounds is nowadays more prominent than ever. Further studies will be needed to better understand the exact mechanism of linearol on glioblastoma treatment and our preliminary observations need to be validated in glioma xenograft models.

## 4. Materials and Methods

### 4.1. Isolation and Identification of Linearol

Linearol (in highly crystalline form) was derived from the hexane extract of *Sideritis sipylea* Boiss. (collected from the wild from Mt. Kerkis in Samos, Greece). The plant was taxonomically identified by Dr. Nikos Krigas and a voucher specimen has been deposited at the Herbarium of the School of Pharmacy, Aristotle University of Thessaloniki, as well as another one stored in the Herbarium BBGK of the Balkan Botanic Garden of Kroussia, Institute of Plant Breeding and Genetic Resources, Hellenic Agricultural Organization Demeter (Athens, Greece). The hexane extract of the aerial parts of the plant was examined in order to identify the presence of diterpenes. In brief, air-dried aerial parts of the plant material (479.5 g) were finely grounded and extracted repeatedly at room temperature with 2 L of hexane (three times) for at least 48 h each time. Chromatographic methods such as CC (Column Chromatography), TLC (Thin Layer Chromatography) and VLC (Vacuum Layer Chromatography) were selected for the isolation of linearol from the extract. The hexane residue (7.74 g) was subjected to VLC (10.0 × 7.0 cm) on silica gel (Merck 60H, Art. 7736), using hexane (He)–ethyl acetate (EtOAc) and EtOAc–methanol mixtures of increasing polarity as eluents to give eighteen fractions of 300 mL each (A-S). Fraction O (eluted with He:EtOAc 10:90, 173.3 mg) was subjected to CC on silica gel (20.0 × 3.5 cm, Merck, Art. 9385, MERCK GLOBAL, Athens, Greece) with dichloromethane (DM)-EtOAc and yielded ten fractions (OA-OK). Fractions OF (eluted with DM:EtOAC 70:30, 15.2 mg) and OG (eluted with DM:EtOAC 60:40, 28.9 mg) were identified as the compound linearol. TLC was used to control the quality of the fractions. For the TLC, a silica gel (Kieselgel F254, Merck, Art. 5554, MERCK GLOBAL, Athens, Greece) stationary phase on aluminum foil (20 × 20 cm, 0.1 mm) with a fluorescence marker was used. The development of the TLC plates was carried out using mixtures of solvents appropriate for each group of fractions. Finally, the TLC plates were sprayed with vanillin-H2SO4 (1:1) [46]. The identification/verification of linearol was performed via 1D and 2D NMR (nuclear magnetic resonance) studies (1H, 13C, gDQCOSY and gHSQCAD). The 1H-NMR and 13C-NMR spectra were recorded in CD3OD using AGILENT DD2 500 (500.1 MHz for 1H-NMR and 125.5 MHz for 13C-NMR) spectrometer. Chemical shifts are reported in δ (ppm) values relative to TMS (Tetramethylsilane) (3.31 ppm for 1H-NMR and 49.05 ppm for 13C-NMR for CD3OD). The data of isolated and identified linearol were compared with those of samples from our collection and/or by a comparison with data reported in the literature (Table S1 and Figures S1–S4) [47].

### 4.2. Cell Lines and Treatment Conditions

The human glioma U87 cell line was obtained from Dr W.K. Alfred Yung (Department of Neuro-Oncology, M.D. Anderson Cancer Center, Houston, TX, USA) and the human glioma cell line T98 was obtained from ATCC (Manassas, VA, USA). Both cell lines were cultured in Dulbecco’s Modified Eagle’s Medium (Gibco BRL, Life Technologies, Grand Island, NY, USA), supplemented with 10% fetal bovine serum (FBS) and 1% penicillin–streptomycin (100 µg/mL of streptomycin, and 100 Units/mL of penicillin), Gibco BRL. The cell lines were incubated in a humidified atmosphere adjusted at 5% CO_2_ and 37 °C. Linearol was isolated from the hexane extract of *Sideritis sipylea*, as described in Section 2.1. Linearol was dissolved in DMSO to a stock concentration of 14.34 mM and stored at −4 °C. Before every experiment, linearol was diluted from the stock solution to the final concertation with culture medium. Less than 1% of DMSO was present in the final volume of each experiment. Cultures of T98 or U87 glioma cells were treated with linearol alone or in combination with radiotherapy (2 or 4Gy).

### 4.3. Viability Assay

In order to assess cell viability, cultures of human glioma cells were treated with linearol in concentrations of 20, 60, 100, 140, 180, 200, 500, 800, 1000 for both the U87 and T98 cell lines. In order to evaluate the possible cytotoxic effects of linearol, we used the Trypan Blue Exclusion assay, as mentioned in our previous experiments [48]. The Trypan Blue Exclusion assay was performed in 24-well plates where 10,000 cells were seeded and after 24 h were exposed to increasing concentrations of linearol. Cell viability was determined at 72 h post-treatment with the use of phase contrast microscopy. The cytotoxicity assay was performed three times and the results presented are the mean of the three. The Trypan Blue Exclusion assay was also performed to determine cell viability after co-treatment with linearol and radiotherapy. 

### 4.4. Crystal Violet Assay 

Crystal violet assay is another cell viability assay that relies on staining cells that are attached to cell culture plates. It was used to further determine cell proliferation in both U87 and T98 cell lines after exposure to two linearol concentrations (91 and 182 μΜ for the T98 cell line, and 98 and 196 μΜ for the U87 cell line). Cells were seeded at a density of 10^5^ per well in 6-well plates and linearol was added in increased concentrations after 24 h. The cells were incubated for 72 h, washed twice with phosphate-buffered saline (PBS) and then Crystal Violet Solution 0.2% (0.2 g Crystal Violet Powder, MERCK in 80 mL ddH2O and 20 mL Methanol, MERCK GLOBAL, Athens, Greece) was added in each well and the plates were placed on a rocking shaker for 2–3 min. The plates were then rinsed with running water and left overnight to dry. Phase-contrast microscopy was used to take pictures of every well-plate the following day.

### 4.5. Flow Cytometric Analysis of DNA Cell Cycle

A total of 10^4^ cells were treated with 91 and 182 μΜ for the T98 cell line, and 98 and 196 μΜ for the U87 cell line of linearol. Untreated cells were used as negative control having less than 1% of DMSO. At least three independent experiments were performed, and all samples were run in triplicates. Cells were treated with linearol at its IC50 and 2IC50 values. Flow cytometric analysis was performed 72 h post-treatment with linearol. For the DNA cell cycle, cells were treated with trypsin, centrifuged, washed with PBS twice and then incubated with PI (Propidium Iodide) working solution (50 µg/mL PI, 20 mg/mL RNase A, and 0.1% Triton X-100, Sigma-Aldrich, St. Louis, MO, USA) for 15 min at 37 °C in the dark. With the use of a flow cytometer (Omnicyt Flow Cytometer, Cytognos, Athens, Greece), the PI fluorescence of 10^4^ individual nuclei was determined. Then, the fractions of cells in G0/G1, S, G2/M and sub-G0/G1 phase were analyzed.

### 4.6. Combination Treatment with Linearol and Radiation

U87 and T98 cells were cultured in 24-well plates and after 24 h were treated with different concentrations of either linearol alone or a combination of linearol and radiation. Radiation was given 2 h after treatment with linearol. Cell viability for every combination was measured using the Trypan Blue Exclusion assay at 72 h post-treatment. The existence of synergy between linearol and radiation was evaluated using the combination index method of Chou and Talalay [49]. Linearol was used in concentrations of 45.5, 68, 91 and 182 μΜ for the T98 cell line and at concentrations of 49, 73.5, 98 and 196 μΜ for the U87 cell line. Two different doses of irradiation were used in both cell lines, 2 and 4 Gy. Eight different combinations with three replicates per condition were used for both series. The affected fraction (Fa) of cells after treatment with linearol alone, irradiation alone or different combinations of those two was calculated and the dose–effect curves were generated. The Combination Index (CI) was determined using CompuSyn software. The CI value represents the effect of combination treatment. CI < 1 is considered synergistic, CI = 1 is considered additive, whereas CI > 1 is considered antagonistic [50].

### 4.7. Scratch Wound Healing Assay

U87 and T98 cells were seeded in 6-well plates at a concentration of 10^5^ cells per well and incubated in 10% FBS-containing medium for 24 h. The cells were then placed in 1% FBS-containing medium for another 24 h (starving period). They were then washed twice with PBS and fresh medium containing 1% FBS was added to each well. The scratch wound was made by scratching each well with a 200 µL sterile pipette tip from top to bottom. Linearol was then added in increased concentrations (91 and 182 μΜ for the T98 cell line, and 98 and 196 μΜ for the U87 cell line). Images of the cells that migrated into the cell-free scratch wound area were acquired using phase contrast microscopy at 0, 24, 48 and 72 h post-treatment. The distance between the wound edges was measured using Image J software version 1.53t (National Institutes of Health, Bethesda, MD, USA).

### 4.8. Statistical Analysis

All results are presented as the mean ± standard deviation. In order to determine the IC50 value of linearol, we used GraphPad Prism software (v. 8.0.0, San Diego, CA, USA, Trial Version) through non-linear regression analysis. Statistical analysis was performed using the SPSS software (IBM SPSS Statistics, Version 28.0.1.0) and determined by a two-tailed Student’s *t*-test. Differences were considered significant when *p* < 0.05. ImageJ software was used to measure the distance between the edges of the scratches.

## Supplementary Materials

The following supporting information can be downloaded at: https://www.mdpi.com/article/10.3390/ijms24043760/s1.

## References

[1] Di Martino E., Smith L., Bradley S.H., Hemphill S., Wright J., Renzi C., Bergin R., Emery J., Neal R.D. (2022). Incidence trends for twelve cancers in younger adults—A rapid review. Br. J. Cancer.

[2] Miranda-Filho A., Piñeros M., Soerjomataram I., Deltour I., Bray F. (2017). Cancers of the brain and cns: Global patterns and trends in incidence. Neuro Oncol..

[3] Jovčevska I., Kočevar N., Komel R. (2013). Glioma and glioblastoma—How much do we (not) know?. Mol. Clin. Oncol..

[4] Alexiou G.A., Tsamis K.I., Vartholomatos E., Peponi E., Tzima E., Tasiou I., Lykoudis E., Tsekeris P., Kyritsis A.P. (2015). Combination treatment of TRAIL, DFMO and radiation for malignant glioma cells. J. Neurooncol..

[5] Davis M.E. (2016). Glioblastoma: Overview of Disease and Treatment. Clin. J. Oncol. Nurs.

[6] Cragg G.M., Newman D.J. (2005). Plants as a source of anti-cancer agents. J. Ethnopharmacol..

[7] Bonafé G.A., Boschiero M.N., Sodré A.R., Ziegler J.V., Rocha T., Ortega M.M. (2022). Natural Plant Compounds: Does Caffeine, Dipotassium Glycyrrhizinate, Curcumin, and Euphol Play Roles as Antitumoral Compounds in Glioblastoma Cell Lines?. Front. Neurol..

[8] Zoi V., Galani V., Vartholomatos E., Zacharopoulou N., Tsoumeleka E., Gkizas G., Bozios G., Tsekeris P., Chousidis I., Leonardos I. (2021). Curcumin and Radiotherapy Exert Synergistic Anti-Glioma Effect In Vitro. Biomedicines.

[9] Persano F., Gigli G., Leporatti S. (2022). Natural Compounds as Promising Adjuvant Agents in The Treatment of Gliomas. Int. J. Mol. Sci..

[10] González-Burgos E., Carretero M.E., Gómez-Serranillos M.P. (2011). *Sideritis* spp.: Uses, chemical composition and pharmacological activities—A review. J. Ethnopharmacol..

[11] Plants of the World Online. http://www.plantsoftheworldonline.org/taxon/urn:lsid:ipni.org:names:21227-1.

[12] Tadić V.M., Jeremic I., Dobric S., Isakovic A., Markovic I., Trajkovic V., Bojovic D., Arsic I. (2012). Anti-inflammatory, gastroprotective, and cytotoxic effects of *Sideritis scardica* extracts. Planta Med..

[13] Jeremic I., Tadic V., Isakovic A., Trajkovic V., Markovic I., Redzic Z., Isakovic A. (2013). The mechanisms of in vitro cytotoxicity of mountain tea, *Sideritis scardica*, against the C6 glioma cell line. Planta Med..

[14] Ambrosio S.R., Furtado N.A., de Oliveira D.C., da Costa F.D., Martins C.H.G., de Carvalho T.C., Porto T.S., Veneziani R.C.S. (2008). Antimicrobial activity of kaurane diterpenes against oral pathogens. Z. Nat. C J. Biosci..

[15] Hwang B.Y., Lee J.H., Koo T.H., Kim H.S., Hong Y.S., Ro J.S., Lee K.S., Lee J.J. (2001). Kaurane diterpenes from *Isodon japonicus* inhibit nitric oxide and prostaglandin E2 production and NF-kappaB activation in LPS-stimulated macrophage RAW264.7 cells. Planta Med..

[16] de las Heras B., Hortelano S., Girón N., Bermejo P., Rodríguez B., Boscá L. (2007). Kaurane diterpenes protect against apoptosis and inhibition of phagocytosis in activated macrophages. Br. J. Pharmacol..

[17] Kilic T., Topcu G., Goren A.C., Aydogmus Z., Karagoz A., Yildiz Y.K., Aslan I. (2020). Ent-kaurene Diterpenoids from *Sideritis lycia* with Antiviral and Cytotoxic Activities. Rec. Nat. Prod..

[18] Castrillo A., de las Heras B., Hortelano S., Rodriguez B., Villar A., Bosca L. (2001). Inhibition of the nuclear factor kB (NF-kB) pathway by tetracyclic kaurene diterpenes in macrophages. Specific effects on NF-kB-inducing kinase activity and on the coordinate activation of ERK and p38 MAPK. J. Biol. Chem..

[19] Stupp R., Mason W.P., van den Bent M.J., Weller M., Fisher B., Taphoorn M.J.B., Belanger K., Brandes A.A., Marosi C., Bogdahn U. (2005). Radiotherapy plus Concomitant and Adjuvant Temozolomide for Glioblastoma. N. Engl. J. Med..

[20] Yabo Y.A., Niclou S.P., Golebiewska A. (2022). Cancer cell heterogeneity and plasticity: A paradigm shift in glioblastoma. Neuro Oncol..

[21] Ma R., Taphoorn M.J.B., Plaha P. (2021). Advances in the management of glioblastoma. J. Neurol. Neurosurg. Psychiatry.

[22] Sestito S., Runfola M., Tonelli M., Chiellini G., Rapposelli S. (2018). New Multitarget Approaches in the War Against Glioblastoma: A Mini-Perspective. Front. Pharmacol..

[23] Akter R., Najda A., Rahman H., Shah M., Wesołowska S., Hassan S.S.U., Mubin S., Bibi P., Saeeda S. (2021). Potential Role of Natural Products to Combat Radiotherapy and Their Future Perspectives. Molecules.

[24] Zoi V., Galani V., Tsekeris P., Kyritsis A.P., Alexiou G.A. (2022). Radiosensitization and Radioprotection by Curcumin in Glioblastoma and Other Cancers. Biomedicines.

[25] Bellettato C.M., Scarpa M. (2018). Possible strategies to cross the blood–brain barrier. Ital. J. Pediatr..

[26] Patrignani F., Prasad S., Novakovic M., Marin P.D., Bukvicki D. (2021). Lamiaceae in the treatment of cardiovascular diseases. Front. Biosci..

[27] Khoury M., Stien D., Eparvier V., Ouaini N., Beyrouthy M.E.L. (2016). Report on the Medicinal Use of Eleven Lamiaceae Species in Lebanon and Rationalization of Their Antimicrobial Potential by Examination of the Chemical Composition and Antimicrobial Activity of Their Essential Oils. Evid.-Based Complement. Altern. Med..

[28] Bekut M., Brkić S., Kladar N., Dragović G., Gavarić N., Božin B. (2018). Potential of selected Lamiaceae plants in anti(retro)viral therapy. Pharmacol. Res..

[29] Raja R.R. (2012). Medicinally potential plants of Labiatae (Lamiaceae) family: An overview. Res. J. Med. Plants..

[30] World Health Organization (2013). WHO Traditional Medicine Strategy: 2014–2023.

[31] Natsume A., Ishii D., Wakabayashi T. (2005). IFN-beta down-regulates the expression of DNA repair gene MGMT and sensitizes resistant glioma cells to temozolomide. Cancer Res..

[32] Alonso M.M., Gomez-Manzano C., Bekele B.N., Yung W.K., Fueyo J. (2007). Adenovirus-based strategies overcome temozolomide resistance by silencing the O6-methylguanine-DNA methyltransferase promoter. Cancer Res..

[33] Montaldi A.P., Godoy P.R., Sakamoto-Hojo E.T. (2015). APE1/REF-1 down-regulation enhances the cytotoxic effects of temozolomide in a resistant glioblastoma cell line. Mutat. Res. Genet. Toxicol. Environ. Mutagen..

[34] Puliyappadamba V.T., Hatanpaa K.J., Chakraborty S., Habib A.A. (2014). The role of NF-κB in the pathogenesis of glioma. Mol. Cell Oncol..

[35] Nogueira L., Ruiz-Ontañon P., Vazquez-Barquero A., Lafarga M., Berciano M.T., Aldaz M., Grande L., Casafont I., Segura V., Robles E.F. (2011). Blockade of the NFκB pathway drives differentiating glioblastoma-initiating cells into senescence both in vitro and in vivo. Oncogene.

[36] Robe P.A., Bentires-Alj M., Bonif M., Rogister B., Deprez M., Haddada H., Khac M.T., Jolois O., Erkmen K., Merville M.P. (2004). In vitro and in vivo activity of the nuclear factor-kappaB inhibitor sulfasalazine in human glioblastomas. Clin. Cancer Res..

[37] Wang H., Wang H., Zhang W., Huang H.J., Liao W.S., Fuller G.N. (2004). Analysis of the activation status of Akt, NFkappaB, and Stat3 in human diffuse gliomas. Lab. Invest..

[38] Levin V., Maor M., Thall P., Yung W., Bruner J., Sawaya R., Kyritsis A., Leeds N., Woo S., Rodríguez L. (1995). Phase II study of accelerated fractionation radiation therapy with carboplatin followed by vincristine chemotherapy for the treatment of glioblastoma multiforme. Int. J. Radiat. Oncol. Biol. Phys..

[39] Lee H.C., Kim D.W., Jung K.Y., Park I.C., Park M.J., Kim M.S., Woo S.H., Rhee C.H., Yoo H., Lee S.H. (2004). Increased expression of antioxidant enzymes in radioresistant variant from U251 human glioblastoma cell line. Int. J. Mol. Med..

[40] Olivier C., Oliver L., Lalier L., Vallette F.M. (2021). Drug Resistance in Glioblastoma: The Two Faces of Oxidative Stress. Front. Mol. Biosci..

[41] Traverso N., Ricciarelli R., Nitti M., Marengo B., Furfaro A.L., Pronzato M.A., Marinari U.M., Domenicotti C. (2013). Role of glutathione in cancer progression and chemoresistance. Oxid. Med. Cell Longev..

[42] González-Burgos E., Carretero M.E., Gómez-Serranillos M.P. (2013). Involvement of Nrf2 signaling pathway in the neuroprotective activity of natural kaurane diterpenes. Neuroscience.

[43] González-Burgos E., Duarte A.I., Carretero M.E., Moreira P.I., Gómez-Serranillos M.P. (2016). Kaurane diterpenes as mitochondrial alterations preventive agents under experimental oxidative stress conditions. Pharm. Biol..

[44] González-Burgos E., Carretero M.E., Gómez-Serranillos M.P. (2013). In vitro permeability study of CNS-active diterpenes from *Sideritis* spp. using cellular models of blood-brain barrier. Planta Med..

[45] Franken N.A., Rodermond H.M., Stap J., Haveman J., van Bree C. (2006). Clonogenic assay of cells in vitro. Nat. Protoc..

[46] Stahl E. (1969). Thin-Layer Chromatography.

[47] Baser K.H.C., Bondi M.L., Bruno M., Kirimer N., Piozzi F., Tumen G., Vassallo N. (1996). An ent-kaurane from *Sideritis huber-morathii*. Phytochemistry.

[48] Kastamoulas M., Chondrogiannis G., Kanavaros P., Vartholomatos G., Bai M., Briasoulis E., Arvanitis D., Galani V. (2013). Cytokine effects on cell survival and death of A549 lung carcinoma cells. Cytokine.

[49] Chou T.C. (2006). Theoretical Basis, Experimental Design, and Computerized Simulation of Synergism and Antagonism in Drug Combination Studies. Pharmacol. Rev..

[50] Chou T.C. (2010). Drug Combination Studies and Their Synergy Quantification Using the Chou-Talalay Method. Cancer Res..

