# Supplementary material for: Therapeutic Potential of Linearol in Combination with Radiotherapy for the Treatment of Glioblastoma In Vitro
Source: Int J Mol Sci. 2023 Feb 13;24(4):3760. doi: 10.3390/ijms24043760 (PMC9958920; doi:10.3390/ijms24043760)
Supplement: Supplementary file 1 [file ijms-24-03760-s001.zip › ijms-2141804-supplementary.pdf]

SUPPLAMENTARY MATERIAL

Therapeutic potential of Linearol in combination with radiotherapy for the treatment of glioblastoma *in vitro*

Table S1. ^{1}H and ^{13}C NMR of linearol (CD_3OD, 500 MHz)

No	δ_C	Type C	δ_H	H	J (Hz)
1	38.0	CH_2	2.00-1.50	2	o.s
2	26.2	CH_2	2.00-1.50	2	o.s
3	71.3	CH-OH	3.60	1	o.s
4	38.3	C	-	-	-
5	38.4	CH	2.00-1.50	1	o.s
6	16.9	CH_2	2.00-1.50	2	o. s
7	76.4	CH-OH	3.48	1	br s
8	45.0	C	-	-	-
9	50.2	CH	2.00-1.50	1	o.s
10	38.5	C	-	-	-
11	17.1	CH_2	2.00-1.50	2	d (J=7.5)
12	33.2	CH_2	2.00-1.50	2	d (J=1.0)
13	43.8	CH	2.54	1	br s
14	41.0	CH_2	2.00-1.50	2	o.s
15	43.5	CH_2	2.24	2	br s
16	155.2	C	-	-	-
17a	102.4	CH_2	4.80	1	br s
17b			4.75	1	br s
18a	65.3	CH_2	3.97	1	d (J=11.5)
18b			3.82	1	d (J=11.5)
19	11.3	CH_3	0.73	3	s
20	17.5	CH_3	1.09	3	s
C=O	172.0	OC=O	-	-	-
OCH_3	19.6	CH_3	2.04	3	s

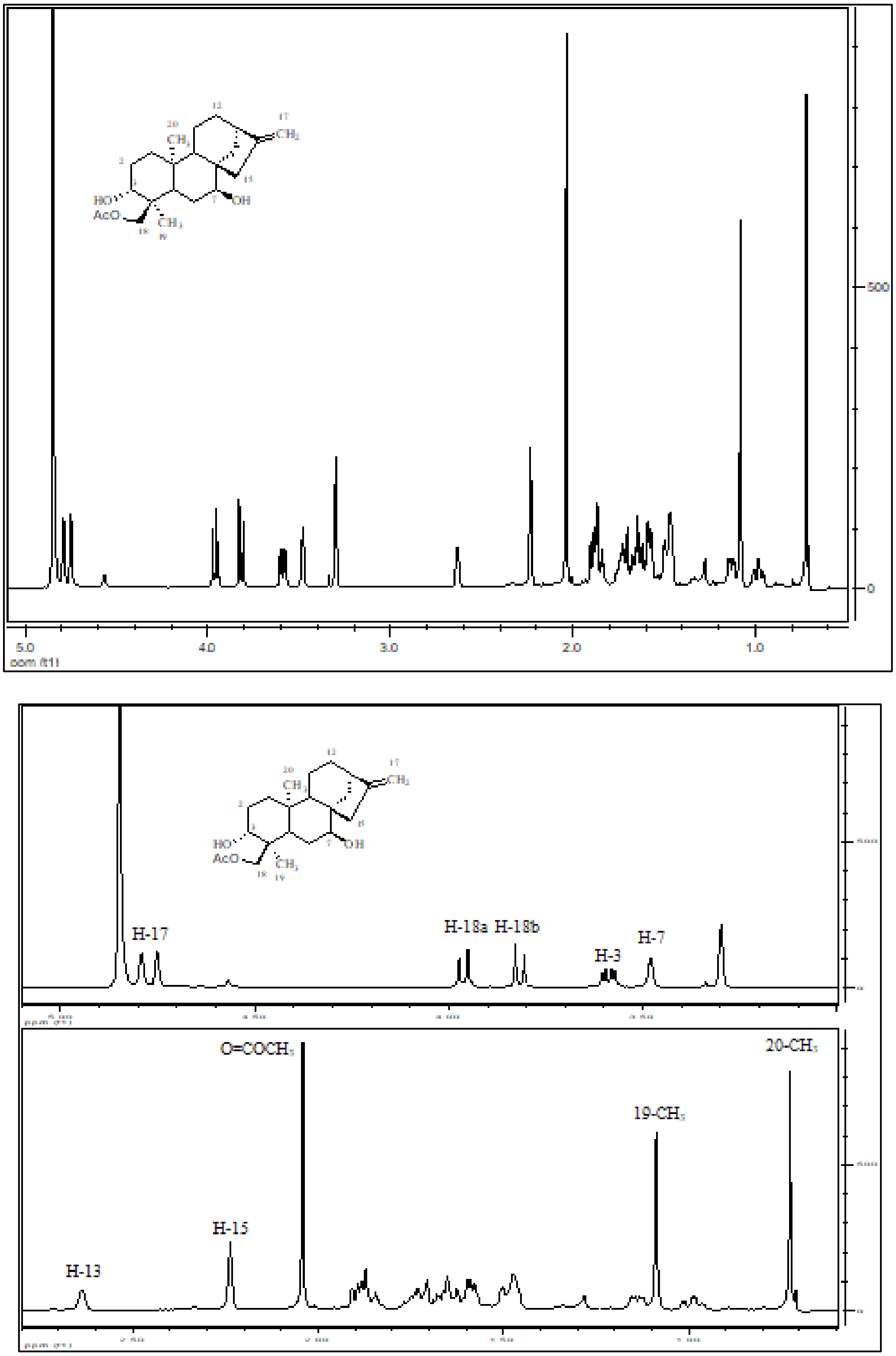

Figure S1. ^{1}H-NMR spectrum of linearol (CD_3OD, 500 MHz)

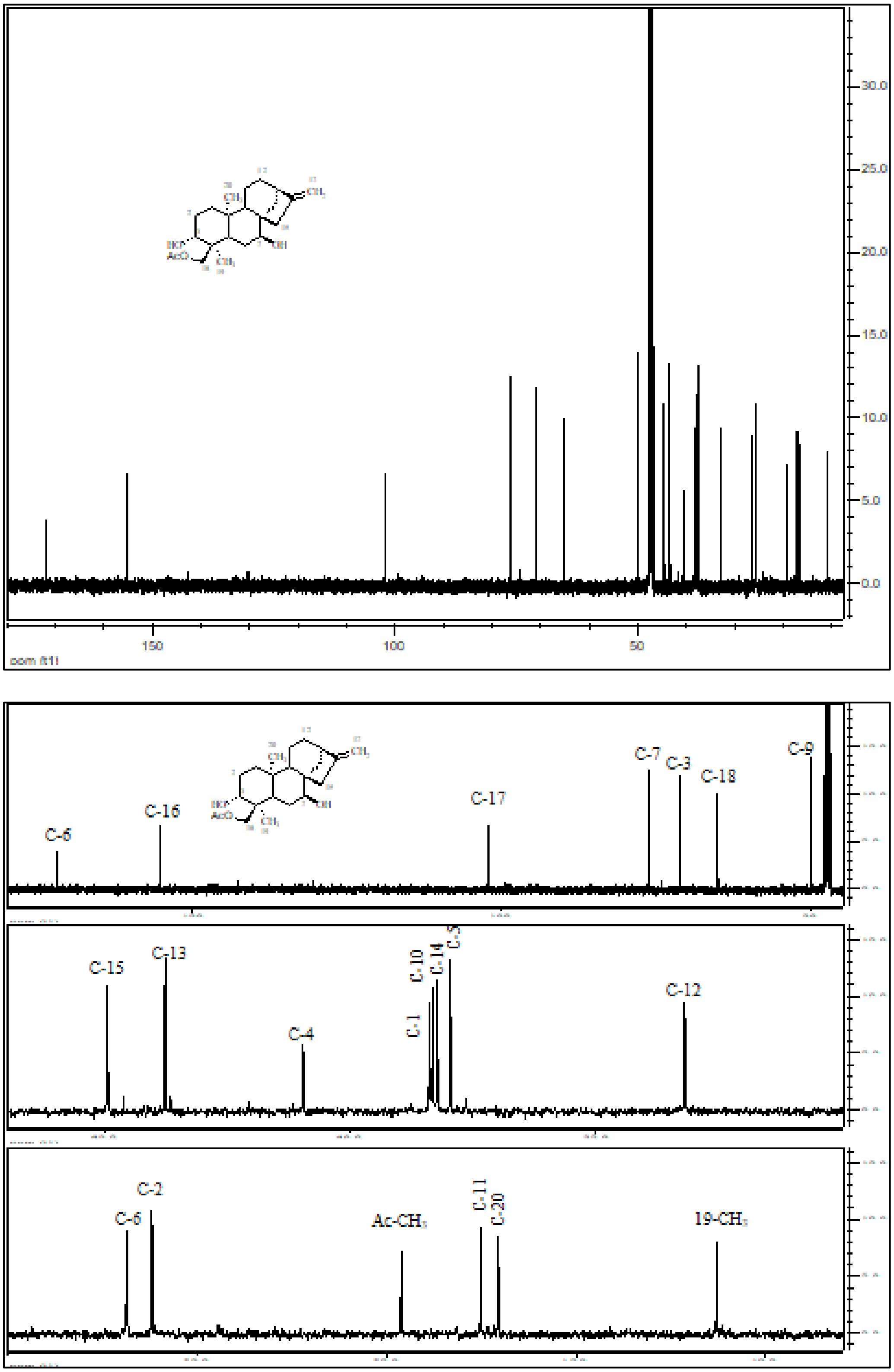

Figure S2. ^{13}C-NMR spectrum of linearol (CD_3OD, 500 MHz)

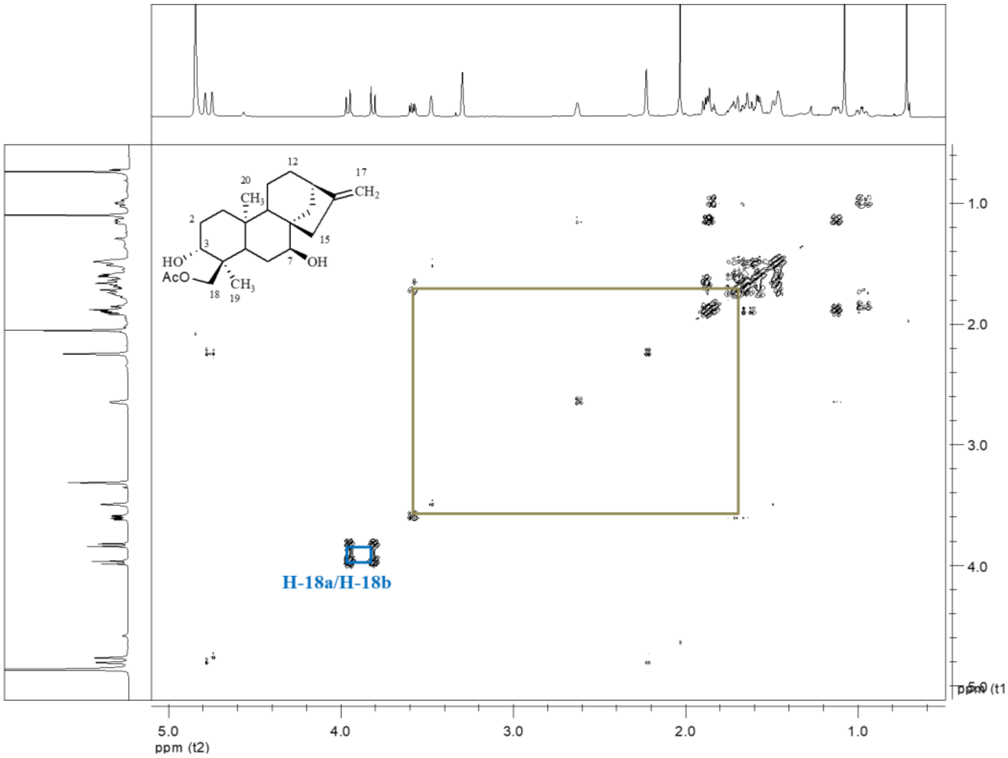

Figure S3. gDQCOSY spectrum of linearol (CD_3OD, 500 MHz)

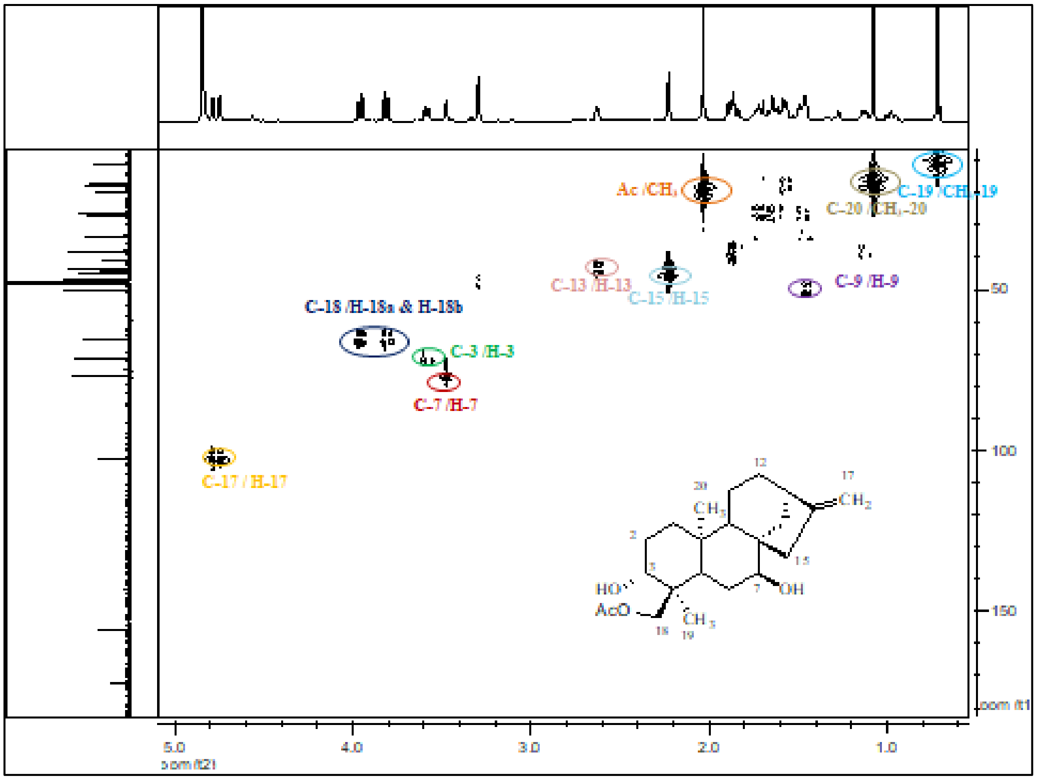

Figure S4. gHSQCAD spectrum of linearol (CD_3OD, 500 MHz)
